# Towards Restoration of Missing Underwater Forests

**DOI:** 10.1371/journal.pone.0084106

**Published:** 2014-01-08

**Authors:** Alexandra H. Campbell, Ezequiel M. Marzinelli, Adriana Vergés, Melinda A. Coleman, Peter D. Steinberg

**Affiliations:** 1 Sydney Institute of Marine Sciences, Mosman, NSW, Australia; 2 Centre for Marine Bio-Innovation and School of Biological, Earth and Environmental Sciences, University of New South Wales, Sydney, NSW, Australia; 3 Evolution and Ecology Research Centre, University of New South Wales, Sydney, NSW, Australia; 4 Department of Primary Industries, NSW Fisheries, Coffs Harbour, NSW, Australia; 5 Advanced Environmental Biotechnology Centre, Nanyang Technical University, Singapore, Singapore; University of Sydney, Australia

## Abstract

Degradation of natural habitats due to urbanization is a major cause of biodiversity loss. Anthropogenic impacts can drive phase shifts from productive, complex ecosystems to less desirable, less diverse systems that provide fewer services. Macroalgae are the dominant habitat-forming organisms on temperate coastlines, providing habitat and food to entire communities. In recent decades, there has been a decline in macroalgal cover along some urbanised shorelines, leading to a shift from diverse algal forests to more simple turf algae or barren habitats. *Phyllospora comosa*, a major habitat forming macroalga in south-eastern Australia, has disappeared from the urban shores of Sydney. Its disappearance is coincident with heavy sewage outfall discharges along the metropolitan coast during 1970s and 1980s. Despite significant improvements in water-quality since that time, *Phyllospora* has not re-established. We experimentally transplanted adult *Phyllospora* into two rocky reefs in the Sydney metropolitan region to examine the model that Sydney is now suitable for the survival and recruitment of *Phyllospora* and thus assess the possibility of restoring *Phyllospora* back onto reefs where it was once abundant. Survival of transplanted individuals was high overall, but also spatially variable: at one site most individuals were grazed, while at the other site survival was similar to undisturbed algae and procedural controls. Transplanted algae reproduced and recruitment rates were higher than in natural populations at one experimental site, with high survival of new recruits after almost 18 months. Low supply and settlement success of propagules in the absence of adults and herbivory (in some places) emerge as three potential processes that may have been preventing natural re-establishment of this alga. Understanding of the processes and interactions that shape this system are necessary to provide ecologically sensible goals and the information needed to successfully restore these underwater forests.

## Introduction

Ecosystem degradation is an increasingly common global phenomenon affecting many different types of habitats [1], from terrestrial forests [2] to coral reefs (e.g. in the Caribbean) [3] and is often linked to multiple anthropogenic stressors [4]. Degradation and loss of important habitat-forming organisms, in particular, can have major ecosystem-level consequences, including impacts on local productivity and biodiversity [5]. Typically, losses of habitat-forming species facilitate phase-shifts from complex, productive habitats that provide important services to simpler, less desirable habitats that are less productive and diverse [1]. Phase shifts associated with environmental changes are often described retrospectively, so understanding the mechanisms that caused them can be challenging [2]. Furthermore, phase shifts can persist even after the perturbations that caused them are identified and reduced [6].

Seaweeds (macroalgae) are the “trees” of the oceans, providing habitat structure, food and shelter for other marine organisms [7]. Several species of large canopy forming macroalgae and the habitats they provide are declining in many temperate ecosystems [8], which is alarming given how disturbances to these habitats can impact upon understory benthic community composition [9], associated fish assemblages [10], [11] and trophic food webs [12]. In some places, declines, disappearances and fragmentation of canopy-forming kelps and other seaweeds has been attributed to human impacts, including increased coastal urbanisation [13], [14], ocean warming [15] and overfishing [8], [16]. The loss of habitat-forming seaweeds from reefs and their replacement by smaller, more tolerant algae, sessile invertebrates or barren habitats can lead to phase shifts in coastal marine ecosystems (e.g. decline of *Ecklonia radiata* in South Australia) [14].

In temperate Australia, coastal ecosystems are very species rich [17], with a particularly high degree of endemism in marine algae [18]. Many macroalgae in Australia appear to be declining, range-shifting or disappearing due to ocean warming [15], [19], [20] or urbanisation [14], [21] and *Phyllospora comosa* (Labillardière) C.Agardh (hereafter *Phyllospora*) is a notable example of this phenomenon. *Phyllospora* is a perennial, large (typically 1–2 m long thalli), habitat forming fucoid seaweed with apical growth and dioecious thalli with reproductive conceptacles [22]. Individuals appear to live for more than two years and are reproductive year-round [23]. *Phyllospora* forms extensive underwater forests (often accounting for 100% of canopy cover) [23] on shallow rocky reefs along more than 2500 km of coastline throughout much of temperate Australia (from Robe in South Australia to Port Macquarie in New South Wales) [24]. There is, however, a conspicuous gap in this distribution: *Phyllospora* is absent from the metropolitan coastline of Sydney, Australia's largest city, but persists on reefs north and south of this region [25].

*Phyllospora* was common and abundant on shallow subtidal rocky reefs in Sydney until the late 1970s, when it disappeared from the region and remains locally extinct [25]. ‘Crayweed’ (as *Phyllospora* is known by local recreational fishers) provides resources to a wide variety of organisms, including commercially important species of fish, crayfish and abalone [26]. This seaweed influences local biodiversity in other systems as well. Wrack from detached and decomposing *Phyllospora* is an important source of detritus to soft sediment systems nearby and supports a more diverse assemblage of invertebrates than detritus originating from other macroalgae [27]. Thus, its disappearance has likely had major consequences for local ecosystems.

The disappearance of *Phyllospora* from reefs in Sydney coincided with a peak in high volume, near-shore sewage outfall discharges along the metropolitan coastline during the 1970s and 1980s (Coleman et al. 2008). Although causation has not been formally established, embryos of this species are particularly susceptible to pollutants commonly found in sewage, to the extent that they are used as a test species in standard ecotoxicological assessments [28]. Despite significant improvements in water-quality along the urbanised coast of Sydney since the introduction of deep water sewage outfalls and the decommissioning of near-shore outfalls [29], [30], *Phyllospora* populations have failed to re-establish.

Given its importance to local ecosystems, restoring this alga in an ecologically sensible way is likely to increase primary productivity and the provision of resources needed to enhance biodiversity in Sydney. Whilst the feasibility and economics of restoration of other benthic marine habitats (e.g. coral reefs) is controversial and the subject of on-going research and debate [31]–[33], the relatively fast growth-rates and short life spans of macroalgae make restoration of impacted temperate reefs via transplantation an attractive management option [34]–[36]. Thus, our aim was to assess whether transplanting *Phyllospora* from existing populations back onto Sydney reefs was a useful method for the potential restoration of this important habitat-forming species [37], [38].

We examined the model that the subtidal environment in Sydney is now suitable for the survival and recruitment of *Phyllospora.* We predicted that individuals transplanted into Sydney would survive and perform (see below) similarly to individuals in extant *Phyllospora* habitats and procedural controls. We also predicted similar recruitment levels in and surrounding transplanted patches to that in natural populations. To test these hypotheses, we transplanted healthy adults from extant populations outside of the city back onto Sydney reefs where this species was once common [25]. In addition to survival, we quantified recruitment in restored and naturally occurring (reference) patches of *Phyllospora.* To assess any sublethal impacts of transplantation back onto Sydney reefs, we also quantified algal condition (size, photosynthetic activity and signs of stress or disease such as algal bleaching and epibiosis) during the experiment to test the hypothesis that these variables would be similar on transplanted adults to those in natural populations or procedural controls.

## Materials and Methods

To assess whether *Phyllospora comosa* individuals could survive on rocky reefs within the Sydney metropolitan area, we transplanted adults from two extant populations on the periphery of Sydney (donor habitats) into two physically similar reef habitats within metropolitan Sydney where *Phyllospora* occurred in the past (recipient habitats). The donor populations in the periphery of Sydney were in Cronulla (34°03′23″ S 151°09′23″ E) and Palm Beach (33°35′58″ S 151°19′43″ E). Shallow rocky reefs at these places are characterised by a mosaic of patches of *Phyllospora* forests (size-range: 7–40 m^2^), barrens, turfing corallines and ‘fringe’ habitats [39], with few individuals of the kelp *Ecklonia radiata*. The recipient habitats in Sydney were in Long Bay (33°57′58″ S 151°15′27″ E) and Cape Banks (33°59′57″ S 151°14′52″ E). Reefs at these recipient sites are very similar to those in donor places, except that patches of *Ecklonia* forests are more abundant and *Phyllospora* forests are absent. Collections and transplantations were carried out under a Scientific Collection Permit (# P00/0054-6.0) issued to the authors by the New South Wales Department of Primary Industries (Fishing and Aquaculture). This study did not involve any endangered or protected species. Experiments were done twice. In the first experiment, forty adults were collected haphazardly (collected individuals were typically 1–3 m apart) at the same depth (1–2 m) from each donor habitat by carefully detaching the holdfast from the substratum. Our haphazard sampling meant that we collected a random mix of reproductive and non-reproductive male and female individuals. Individuals were tagged, measured (max. length) and kept in 50 L containers with seawater for ∼2–3 hs during transportation until reattachment.

*Phyllospora* individuals from the two donor habitats were randomly allocated to one of three treatments: i) Transplanted individuals (‘TP’; *n* = 20), moved to recipient habitats in Sydney (from Cronulla to Long Bay or from Palm Beach to Cape Banks); ii) Disturbed individuals (‘D’; *n* = 10), which were disturbed in the same manner as required for transplantation, but were returned to their original donor habitat; iii) Translocated individuals (‘TL’; *n* = 10), which were similarly disturbed, but were taken to a different place where *Phyllospora* naturally occurs (the other donor habitat, *i.e.* from Cronulla to Palm Beach and *vice versa*). Undisturbed individuals (‘U’; *n* = 20) were haphazardly selected and marked *in situ* but otherwise not handled. Disturbance and translocation treatments allowed distinguishing the effects of transplantation to a recipient habitat from the possible effects of the transplantation procedure or the effects of simply moving the algae to a novel place in an otherwise suitable habitat [40], [41]. Fewer replicates of procedural controls were used in this pilot experiment because we wanted to try the transplantation methodology, as this was our first attempt at transplanting *Phyllospora*.

Algae that were removed from the substratum (TP, D and TL individuals) were held in place *via* cable-tie attachment to 0.25 m^2^ plastic meshes, which were 0.5–2 m apart and had been previously attached to bare rock in barren patches ∼ at 1–2 m depth. A mosaic of patches of *Ecklonia*, turfing corallines, ‘fringe’ habitat and other barren patches surrounded these areas. Five individuals were attached to each mesh to approximate natural densities (mean density 6.7± S.E. 1.1 per 0.25 m^2^), creating a patch of ∼4–5 m^2^ at each place. Each individual had one cable-tie loosely fastened around the stipe (directly above the holdfast) and three cable-ties threaded through the former and tied to the mesh to hold the algae in an upright position. During this first experiment (28 February to 9 May 2011), each alga was revisited every 2–4 weeks when we recorded survival and percentage of thallus bleaching (tissue discolouration from deep brown to white) and epibiosis, which are factors that can negatively affect algae [42], [43]. At the end of the experiment, all individuals were collected and their lengths were measured. The maximum photosynthetic quantum yield of 3 individuals from each treatment was measured using a Pulse Amplitude Modulated (PAM) fluorometer (WALZ, Germany). PAM data are frequently used to assess the ecophysiological condition of seaweeds [44]. Algae were dark-adapted for 5 minutes prior to measurement.

The experiment was repeated in late winter/spring (started 9 August 2011). In this second experiment, algae from both donor populations were transplanted to each recipient site to avoid confounding due to possible differences between algae from different sources. Sixty algae were collected from each donor place (see above) and randomly assigned to three treatments: i) individuals TP to Long Bay (*n* = 20), ii) individuals TP to Cape Banks (*n* = 20), iii) TL individuals to the other donor place (procedural control; *n* = 20). U individuals (*n* = 20) were haphazardly selected and marked *in situ* (4 sub-patches of 5 individuals each to resemble replication in the other treatments). Algae were attached to meshes as described above and were revisited every 5–10 weeks for 5 months (17 January 2012) to quantify survival, percentage of bleaching and epibiosis and to measure length. Total patch-sizes ranged between 4–8 m^2^ at each place. The Disturbed (D) procedural control was not included in this experiment because no difference in survival or condition was observed between this and the TL control in the previous experiment in autumn (see *Results*). The TL treatment controls not only for disturbing the individuals as required for the transplantation, but also for moving them to an unfamiliar place (see above), so this treatment was deemed sufficient as a procedural control in the repeat experiment.

In this second transplant experiment, we also quantified densities of *Phyllospora* recruits 6 (23 February 2012) and 12 months (21 August 2012) after transplantation using 0.1 m^2^ quadrats (*n* = 5) at three distances relative to the *Phyllospora* patches: inside, at the edge (within 30 cm of the holdfasts of algae at the periphery of the patch) and 2.5 m away from the patch. Recruits were initially defined as *Phyllospora* individuals smaller than 10 cm in length. Densities of recruits at the restored patch in Long Bay were compared to those at 2–3 reference sites where *Phyllospora* occurs naturally: Cronulla, Palm Beach and Bundeena (BU: 34°04′34″ S 151°10′03″ E). Two patches were haphazardly selected within each reference site. Patch-sizes ranged between 7–40 m^2^. Maximum length of recruits (*n* = 5) was also quantified after 6 months. At the restored Long Bay site, the number of transplanted adults and the density of recruits remaining after 12 and 17 months (18 January 2013) were also recorded. At these two times, no formal comparisons with controls could be made because we were unable to locate most undisturbed individuals, most likely due to fouling of the tags (E.M. Marzinelli, personal observation).

Adult survival at the end of the first experiment was analysed using Chi-square tests because we recorded total numbers of individuals per treatment instead of numbers of individuals per mesh/plot (the replicates) for each treatment. Additionally, we compared slopes of ln-transformed survival of algae from each treatment through time using analysis of covariance (ANCOVA) as per [45]. All other data were analysed using analyses of variance, which were used to examine differences among treatments for survival (second experiment only), length, percentage cover of bleaching and epibiota of adults at the end of the experiments (see Tables and Supporting Information for detailed explanation). Analyses were done using GMAV 5 [37]. Densities and lengths of recruits were compared between restored and reference places by doing asymmetrical analyses of variance. Appropriate *F*-ratios were constructed with Mean Squares (MS) calculated in the Permanova add-on for PRIMER 6 using a similarity matrix based on Euclidean distances [46]. Permanova's *F*-ratios for univariate analyses using Euclidean distances are equal to ANOVA Fisher's *F* statistic, which has a known distribution under the true null hypothesis [43 and references therein]. The *F* distribution was used to obtain *P*-values. Prior to all analyses, the assumption of homogeneity of variance was examined using Cochran's *C* test. When Cochran's test was significant and no transformation was appropriate, the analysis of variance was still done because it is robust to departures from the assumptions [47]. Where significant interaction terms were detected, Student-Newman-Keuls (SNK) comparisons of means were used to determine which treatments differed [47]. Data are available upon request.

## Results

### Survival

**Figure 1:**
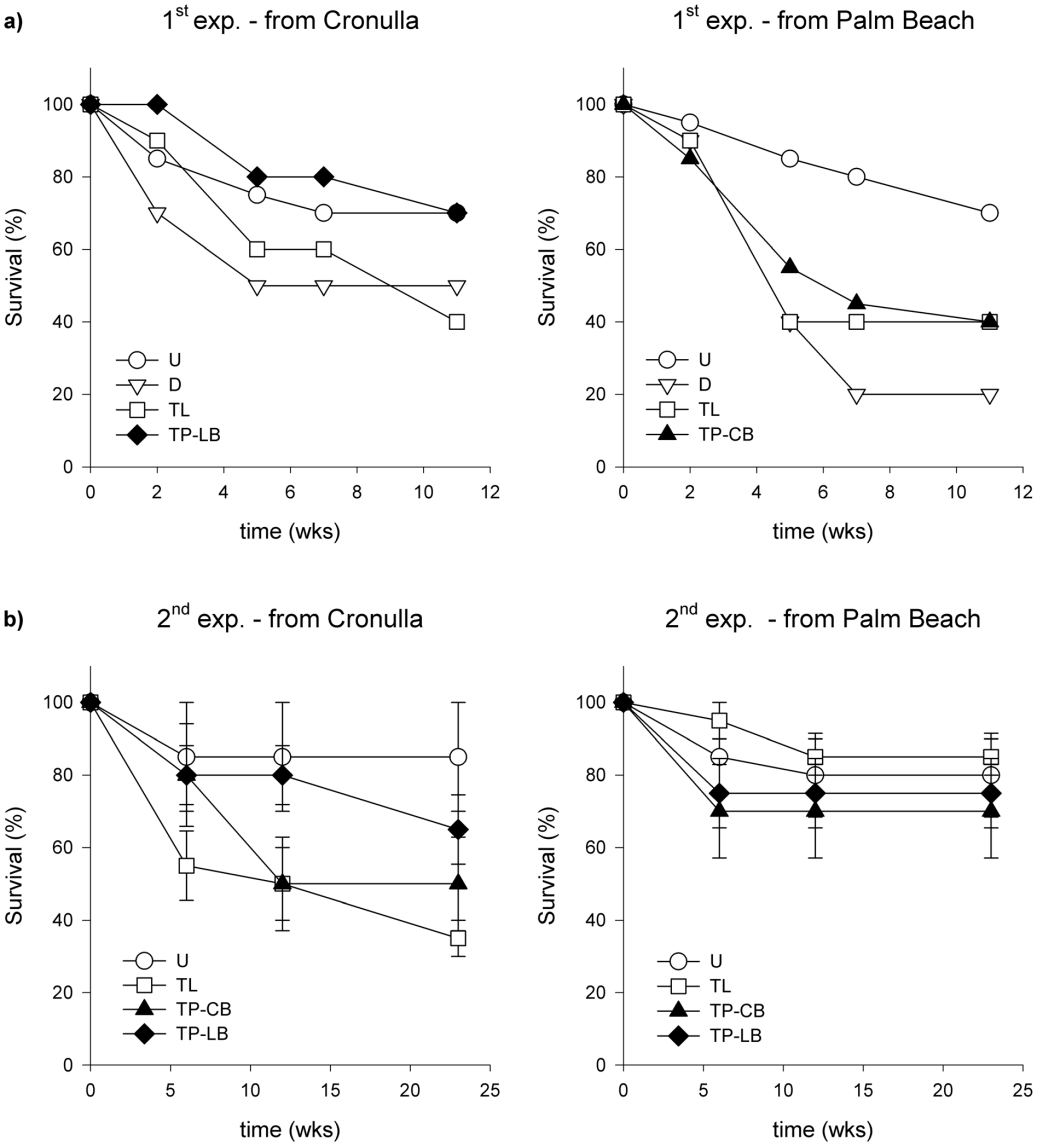
Survival (%) of *Phyllospora* in the (a) first (total for each site) and (b) second (mean ± S.E.; *n* = 4) transplant experiments from Cronulla and Palm Beach to Long Bay and Cape Banks respectively. Treatments are Undisturbed (U; white circles), Disturbed (D; white triangles), Translocated (TL; white squares) and Transplanted to Long Bay (TP-LB; black diamonds) or Cape Banks (TP-CB; black triangles).

In the first experiment, survival of adults transplanted from Cronulla to Long Bay was ∼70%, which did not differ from that of undisturbed individuals or procedural controls (D, TL) in donor habitats (Chi-square  = 3.74, *df* = 3, *P*≥0.29; Fig. 1a). The survival of adults transplanted from Palm Beach to Cape Banks was ∼40%, which was similar to that of algae in procedural controls in donor habitats (20–40%), but lower than the ∼70% survival of undisturbed individuals in the donor habitat (Fig. 1a). This difference was, however, non-significant (Chi-square  = 7.77, *df* = 3, *P*≥0.05). Analyses of survivorship curves showed differences in slope among treatments (from Cronulla: *F*_3,12_ = 3.53, *P*<0.05; from Palm Beach: *F*_3,12_ = 6.86, *P*<0.01), but *a posteriori* contrasts could not resolve where the differences were (Table S1).

Similarly, in the second experiment, survival of transplanted individuals was ∼70–80%, which was similar to that of undisturbed individuals or those in procedural controls in donor habitats (∼80%), despite a trend for lower survival of TL individuals from Cronulla (Table 1, Fig. 1b). Survival of algae originally from different donor populations transplanted to the same recipient site did not differ (ANOVA, *F*_1,13_ = 1.9, *P*≥0.20, Table S2 Fig. 1b). After 12 and 17 months, survival at Long Bay was 50% and 20% respectively. Analyses of differences in slopes of survival curves also indicated no significant overall difference among treatments (from Cronulla: *F*_3,56_ = 2.70, *P*≥0.06; from Palm Beach: *F*_3,56_ = 0.24, *P*≥0.87).

**Table 1:** Analysis of survival (%) of *Phyllospora* five months after the second experimental transplant.

Source	*df*	MS	*F*	*P*
Treatment	3	913	0.85	0.55
Place	1	2813	6.08	**0.02**
Tr x Pl	3	1079	2.33	0.10
Residual	24	463		

Treatment was fixed with 4 levels (U, TL, TP-LB, TP-CB), Place of origin was random with 2 levels (Cronulla, Palm Beach). Replicates were the 0.25 m^2^ plots (*n* = 4). Cochran's test for homogeneity of variances: *C* = 0.24 ns.

### Condition

**Figure 2:**
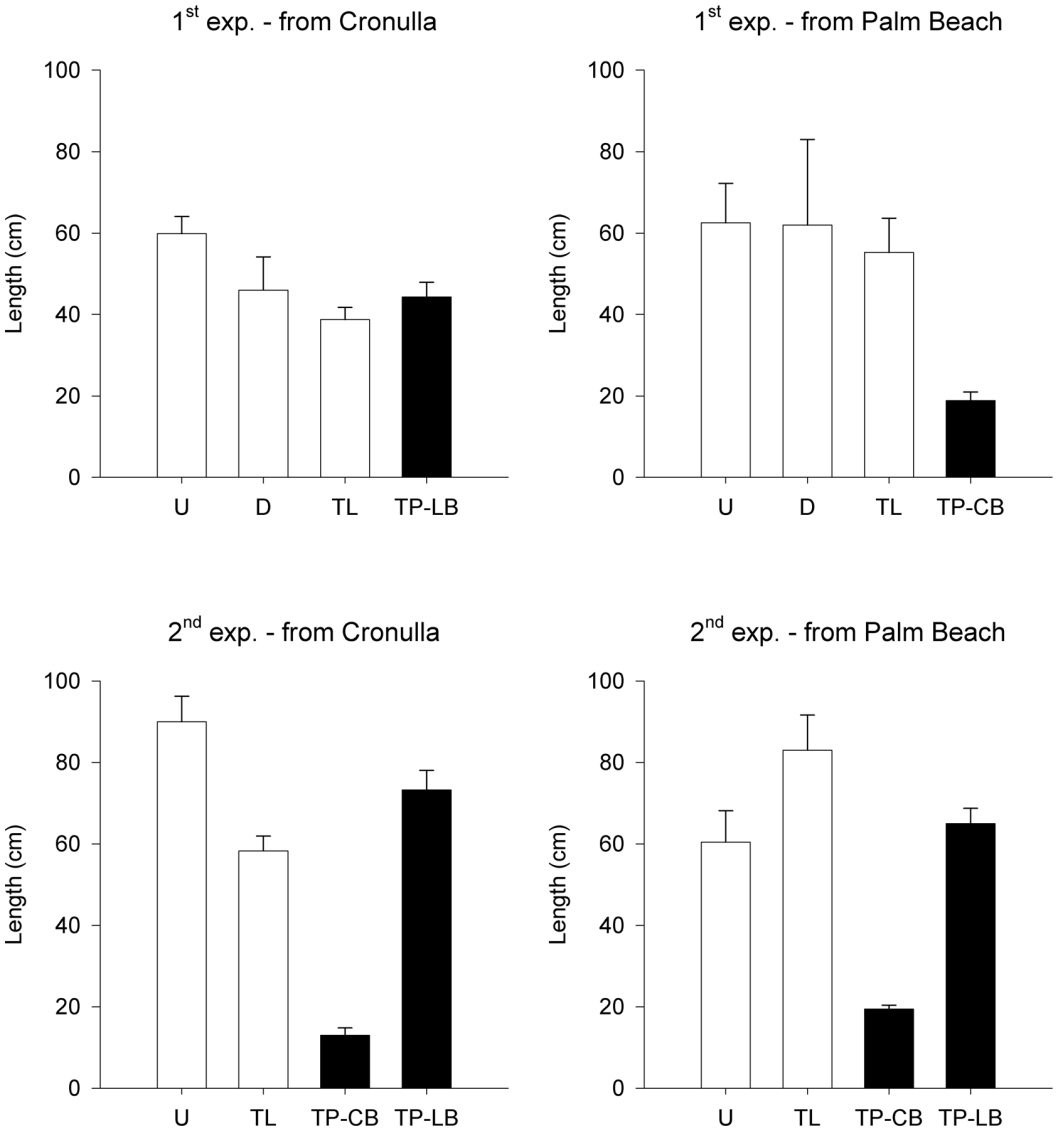
Mean (± S.E.) length of *Phyllospora* at the end of the first (*n* = 3–14) and second (*n* = 7) experimental transplants. Treatments are Undisturbed (U), Disturbed (D), Translocated (TL; white bars) and Transplanted to Long Bay (TP-LB) or Cape Banks (TP-CB; black bars).

In both experiments, the length of individuals transplanted to Cape Banks was 50–70% smaller than that of controls in donor habitats and transplanted individuals at the other recipient site (Table 2, Fig. 2). All individuals at Cape Banks had visible thallus damage consistent with fish bite marks. In the second experiment, there were no differences in length of individuals from different donor populations in the same recipient site, but final length varied significantly between the recipient sites, being ∼70% lower at Cape Banks (ANOVA, *F*_1,24_ = 5.4, *P*<0.03; Figure 2). The mean length of individuals translocated from Cronulla to Palm Beach was ∼25% greater than that of undisturbed individuals in Cronulla and similar to undisturbed individuals at Palm Beach. In contrast, length of translocated individuals from Palm Beach to Cronulla was ∼25% smaller than that of undisturbed individuals in Palm Beach and similar to undisturbed individuals at Cronulla (Table 2, Fig. 2). At the start of both experiments, length of individuals (first experiment: 66.1± S.E. 1.6 cm; second experiment: 59.9± S.E. 1.2 cm) did not differ among treatments (ANOVA; *F*_3,115_ = 0.7, *F*_3,152_ = 0.8 respectively, *P*≥0.5).

**Table 2:** Analyses of length of *Phyllospora* at the end of the (a) first and (b) second experimental transplants.

	(a) Autumn				(b) Spring			
Source	*df*	MS	*F*	*P*	*df*	MS	*F*	*P*
Treatment	3	0.95	1.22	0.44	3	10856	5.87	0.09
Place	1	0.05	0.52	0.48	1	39	0.20	0.66
Tr x Pl	3	0.77	7.95	**<0.01**	3	1848	9.34	**<0.01**
Residual	16	0.10			48	198		
SNK		From Cronulla:				From Cronulla:		
		U = D = TL = TP-LB				U > TL = TP-LB > TP-CB		
		From Palm Beach:				From Palm Beach:		
		U = D = TL > TP-CB				TL > U = TP-LB > TP-CB		

Treatment was fixed with 4 levels (a: U, D, TL, TP; b: U, TL, TP-LB, TP-CB), Place of origin was random with 2 levels (Cronulla, Palm Beach). Replicates were *Phyllospora* individuals (a: *n* = 3; b: *n* = 7). Data in (a) were ln(X+1) transformed. Cochran's test for homogeneity of variances: a: *C* = 0.23 ns; b: *C* = 0.33 ns.

**Figure 3:**
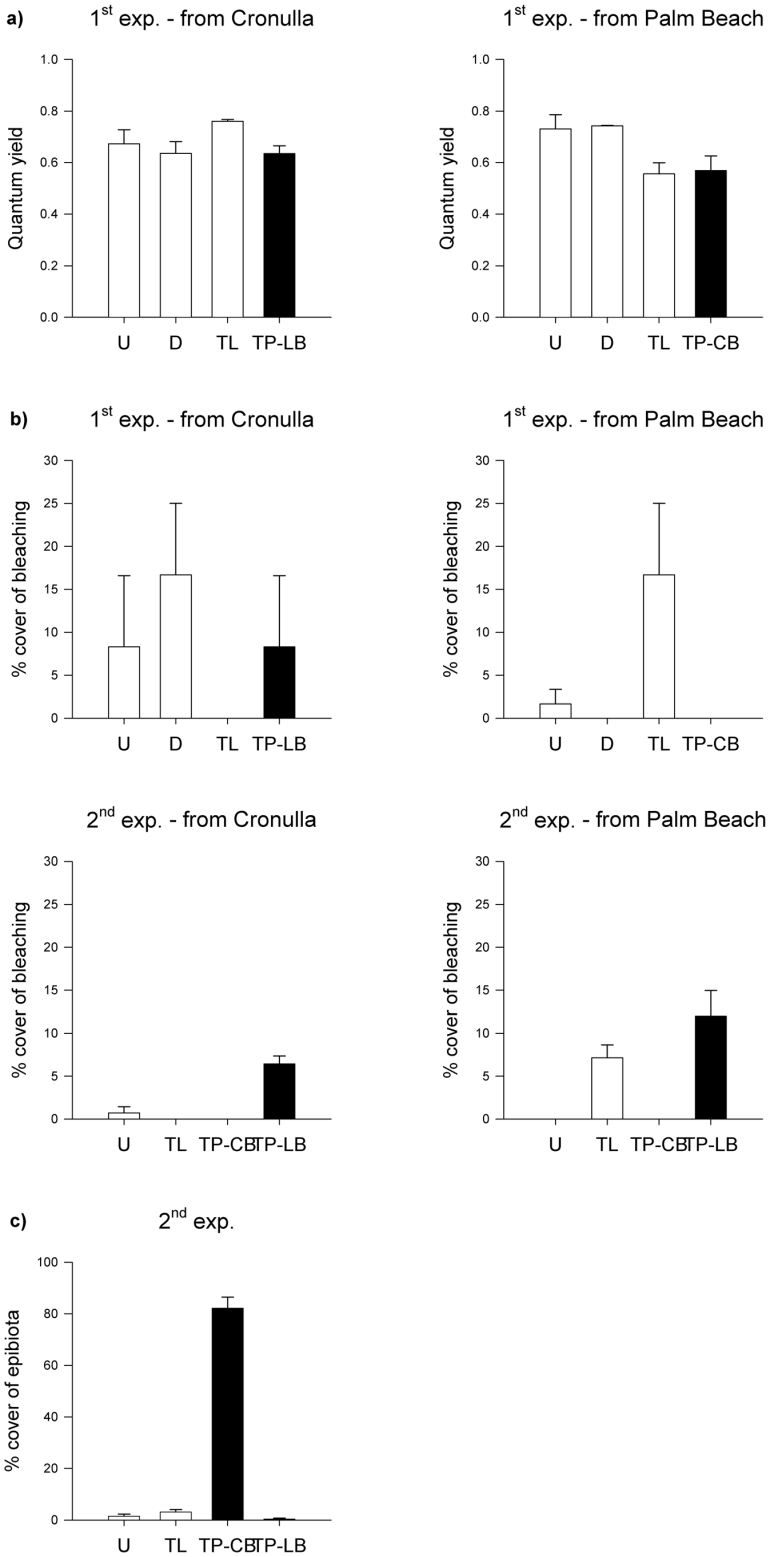
Mean (± S.E.) (a) photosynthetic quantum yield or (b)% cover of bleaching of *Phyllospora* at the end of the first (*n* = 3) and second (*n* = 7) transplant experiment, and (c)% cover of epibiota on *Phyllospora* (*n* = 14) at the end of the second transplant experiment. Treatments are Undisturbed (U), Disturbed (D), Translocated (TL; white bars) and Transplanted to Long Bay (TP-LB) or Cape Banks (TP-CB; black bars).

Mean photosynthetic quantum yield of undisturbed and disturbed algae at Palm Beach was ∼25% higher than that of individuals translocated to Cronulla or those transplanted to the recipient habitat Cape Banks. In contrast, there were no differences in yield among treatments with adults originally from Cronulla (ANOVA, *F*_3,16_ = 5.5, *P*<0.01, Table S3, Fig. 3a).

Percentage cover of thallus bleaching did not differ among treatments in the first experiment (ANOVA, *F*_3,3_ = 0.1, *P*≥0.38, Table S4a, Fig. 3b). However, individuals in Cronulla (U, D, TL from Palm Beach) and those transplanted to Long Bay showed a trend for more bleaching. In the second experiment, individuals transplanted to Long Bay were significantly more bleached than those in other treatments, even after considering bleaching due to translocation effects (ANOVA, *F*_3,48_ = 5, *P*<0.01, Table S4b, Fig. 3b).

At the end of the first experiment, cover of epibiota was ∼0% in all treatments. In the second experiment however, covers of epibiota (mainly filamentous algae) were significantly greater on individuals transplanted to Cape Banks (∼80% *vs* 2%; ANOVA, *F*_3,3_ = 415, *P*<0.01, Table S5, Fig. 3c).

### Recruitment

**Figure 4:**
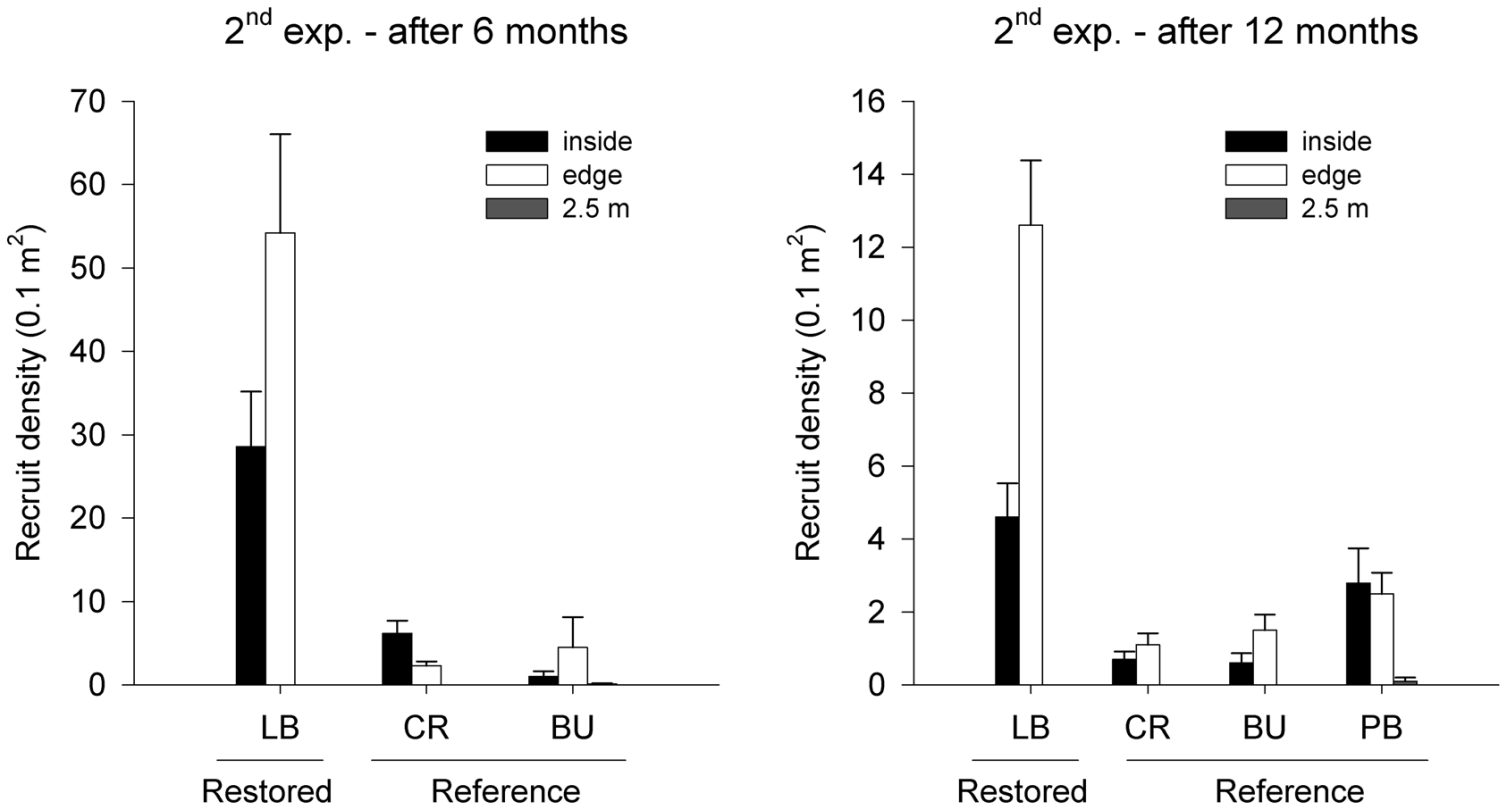
Mean (± S.E.) densities of recruits inside (black bars), at the edge of (∼30 cm from a patch; white bars) or 2.5 m away from (grey bars) patches of *Phyllospora* at the restored place Long Bay (LB; *n* = 5) and at reference places (CR, Cronulla, BU, Bundeena, PB, Palm Beach; *n* = 10) after 6 and 12 months from the start of the second experiment.

**Figure 5:**
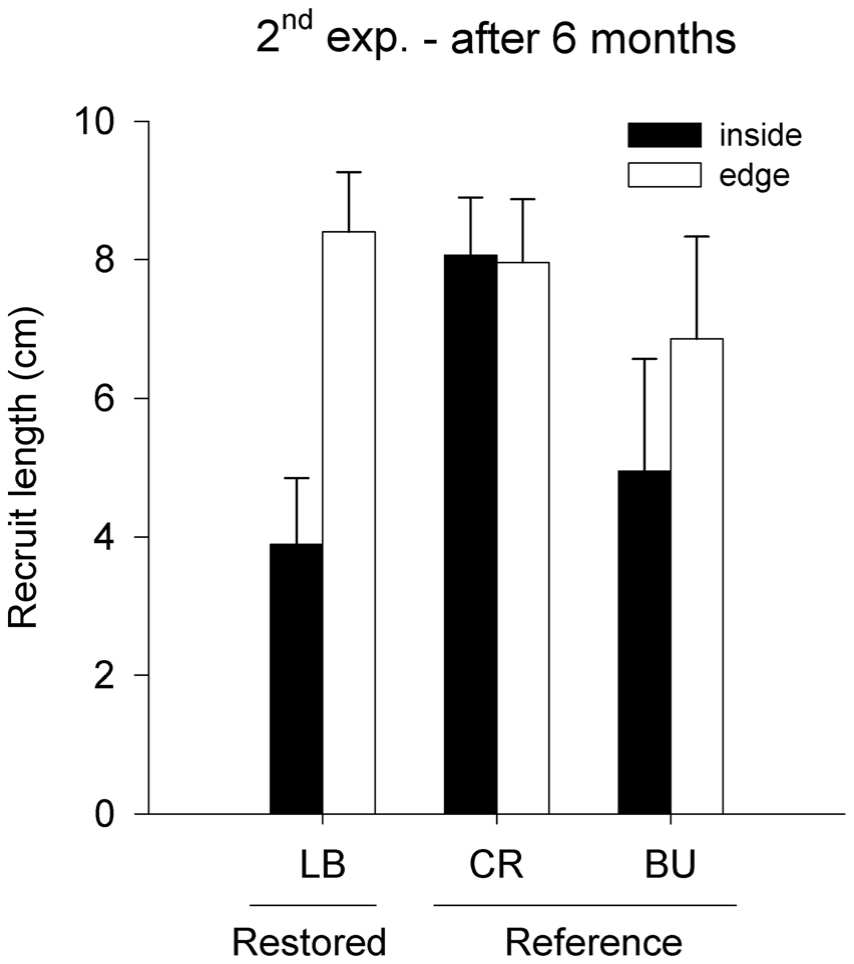
Mean (± S.E.) lengths of recruits inside (black bars) or at the edge (∼30 cm; white bars) of patches of *Phyllospora* at the restored place Long Bay (LB; *n* = 5) and at reference places (CR, Cronulla, BU, Bundeena; *n* = 10) after 6 months from the start of the second experiment.

*Phyllospora* successfully recruited at one recipient site, Long Bay. We counted up to 100 recruits per 0.1 m^2^ in Long Bay 6 months after the start of the experiment. Densities of recruits inside and at the edge of the transplanted patch were significantly greater than those in reference patches (Table 3). In addition, densities of recruits inside the restored patch were greater than at the edge, which, in turn, were greater than those 2.5 m away, where almost no recruitment occurred (Table 3, Fig. 4). No differences in recruitment between distances were found in reference patches, despite a trend for greater densities within and on the edges of patches than further away (Fig. 4). Lengths of recruits did not differ between treatments (ANOVA, *F*_1,43_ = 0.04, *P*≥0.87, Table S6) or distances (ANOVA, *F*_1,43_ = 2.15, *P*≥0.15, Table S6), despite a trend for them to be smaller inside the patch at 2 places (Fig. 5).

**Table 3:** Analyses of densities (0.1 m^2^) of recruits at several distances from restored (R) or reference (Ref) patches of *Phyllospora* (a) six or (b) 12 months after the start of the second experiment.

	(a) 6 months					(b) 12 months			
Source	*df*	MS	*F*		*P*	*df*	MS	*F*	*P*
Distance	2	3327	42		**<0.01**	2	12.74	56	**<0.01**
R vs Ref	1	7651	394		**<0.01**	1	10.97	13	0.07
Place(RvRef)	1	14	1		0.55	2	1.26	4	0.15
D x RvRef	2	2586	33		**<0.01**	2	3.38	15	**<0.01**
Patch(Pl(RvRef))	2	28	0		0.71	3	0.33	1	0.22
D x Pl(RvRef)	2	73	Pooled			4	0.27	Pooled	
D x Pa(Pl(RvRef))	4	25	Pooled			6	0.21	Pooled	
Residual	60	83				84	0.22		
SNK		Inside: R>C					Inside: R>C		
		Edge: R>C					Edge: R>C		
		2.5 m: R = C					2.5 m: R = C		
		R: In > Edge >2.5 m					R: Edge > In >2.5 m		
		Ref: In = Edge = 2.5 m					Ref: In = Edge >2.5 m		

Distance was fixed with 3 levels (inside, edge, 2.5 m), R *vs* Ref was fixed with 2 levels, Place was random nested in RvRef (1 level for R; 2 [5 mo.] or 3 [12 mo.] levels for Ref), Patch was random nested in Place (1 level for R; 2 levels for Ref). The replicates were the quadrats (*n* = 5). Transformation of data in (a) failed to make variances homogeneous; data in (b) were ln(X+1) transformed. Cochran's test for homogeneity of variances: a: *C* = 0.57, *P*<0.01; b: *C* = 0.17 ns. Non-significant terms with *P*>0.25 were pooled.

After 12 months, densities of recruits generally decreased, but were still significantly higher inside and at the edge of the restored patch (where 50% of the transplanted adults still remained) than inside or on the edge of reference patches (Table 3). Densities of recruits 2.5 m away from adult patches did not differ between restored and reference patches (Table 3). Densities at the edge of the restored patch were significantly higher than inside the patch, which, in turn, were higher than those 2.5 m away. In contrast, there were no differences in recruit densities within and on the edge of reference patches, but these were greater than those 2.5 m away (Table 3, Fig. 4). After 17 months, recruit density remained similar to that observed after 12 months (18.6± S.E. 2.5 per 0.1 m^2^). The maximum length of recruits was, however, four times greater than 6 months after the experiment (37.8± S.E. 3.8 cm). Only eight transplanted adults remained attached after 17 months (20% of initial numbers) at Long Bay.

## Discussion

In this study, we transplanted individuals of the habitat forming fucoid alga *Phyllospora comosa* from extant populations into two sites in Sydney where this species has disappeared, but was once abundant. At one site, transplanted individuals survived just as well as conspecifics left undisturbed or in procedural controls and also successfully reproduced. In contrast, individuals transplanted to the other site had lower survival and were in poorer condition than controls. These results suggest that, at some sites, the environment in Sydney is now suitable for the survival and reproduction of *Phyllospora*. Therefore, other processes (such as recruitment limitation and/or herbivory) may be preventing the re-establishment of this seaweed on Sydney reefs. Overall, however, our results are encouraging in the context of potential restoration and ‘re-vegetation’ of impacted, temperate rocky reefs in Australia, where macroalgal flora are highly endemic and in rapid decline [15].

*Phyllospora* individuals transplanted into Long Bay not only survived as well as control algae, but also reproduced successfully and at rates greater than natural populations. After 12 months, the density of recruits on the edge of restored patches was still higher than in natural populations and growth rates were similar, thus supporting the model that this environment is now suitable for the survival and reproduction of *Phyllospora*. After almost 18 months, more than half of the recruits counted initially remained attached to the reef and were almost four times their size at 6 months, suggesting that these individuals could form the beginning of a self-sustaining population of *Phyllospora* at this site, where this species has been missing for more than three decades.

At Cape Banks, although low levels of recruitment were observed after 6 months (data not shown), survival and performance of transplanted adults were much lower than transplanted individuals at Long Bay and control populations, suggesting that ecological processes at this site in particular negatively affected the survival of *Phyllospora*. The length of transplanted individuals was significantly lower than controls after 6 months and all thalli at this site had considerable physical damage consistent with fish bite marks. These observations suggest that herbivory may have led to the poor condition and relatively rapid loss of *Phyllospora* transplants at Cape Banks. Other studies have also highlighted herbivory as one of the key factors limiting success of re-establishment and restoration of declining macroalgal populations [34]. The restored patches of *Phyllospora* may have been too small and represented a novel, concentrated food source with increased vulnerability to herbivores [48] and other processes *via* negative Allee effects [49]. Larger patches may satiate consumers and thus reduce the overall impact of herbivory on the survivors, as occurs in masting events in terrestrial plants [50]. Larger patches of restored *Phyllospora* may thus be more resistant to herbivory and other processes that limit recruitment and survival. Further experiments designed to directly assess the impacts of and determine spatial and temporal variability in herbivory are necessary to understand this potentially important process and assess site suitability prior to commencement of large-scale restoration projects.

Recent population genetics work on *Phyllospora* indicated a high degree of connectivity among populations on the eastern coast of Australia, possibly due to the oceanographic features of the East Australian Current and its associated eddies that may deliver propagules between geographically distant populations (over thousands of kilometres) [23]. In our experiments, recruits were only found within or at the margins of the adult canopy, suggesting that propagule settlement away from the adult canopy is very low (a pattern that is consistent with other fucoids, which generally have limited dispersal (<1 m)) [51], [52]. Thus, even if propagules are delivered to Sydney reefs, the absence of an adult canopy may inhibit recruitment and growth of juveniles. Higher recruitment within or at the edge *versus* outside of adult canopies has been documented in some other brown algae (e.g. the intertidal seaweed *Pelvetia compressa*) [53]. Adult canopies might protect recruits from consumers or competitors [54], [55] or otherwise alter the environment to favour their settlement and survival [56], [57]. Similarly, biotic and/or abiotic differences between sites may have contributed to the higher recruitment rates we observed at Long Bay relative to natural populations of *Phyllospora*. These models of recruitment facilitation in *Phyllospora* need to be tested experimentally in order to provide sound ecological information for sensible restoration and management.

Many examples of declines, range shifts and population fragmentations of canopy-forming macroalgae from temperate reefs are emerging from different places [8], [13], [15], [16]. In many of these examples, the loss of canopy-forming macroalgae leads to phase shifts from complex, productive, biogenic habitats to simpler, less productive and less desirable states. Such phase shifts can be challenging to investigate retrospectively and are often difficult to reverse [58], [59]. Our data support theory and observations from other systems that, once major phase shifts occur, recovery to the initial state can be difficult, even when stressors involved in causing the phase shift initially have been mitigated [16], [58], [59]. In other parts of the world, re-establishment of canopy-forming seaweeds onto reefs where they were formerly dominant has been impeded by competitive exclusion [21], increased herbivory due to overfishing [8], abiotic changes to the environment [60] or combinations of these and other factors [13]. These are all potential reasons why *Phyllospora* has failed to re-establish on Sydney reefs. Our results suggest that low settlement success of propagules may be preventing the natural reestablishment of this habitat-former onto reefs in Sydney, potentially also combined with (at some sites) high rates of herbivory.

Most of the world's ecosystems have been degraded by human activities and the few systems that are still “pristine” are arguably at very high risk of degradation [61]. Despite in many cases extensive conservation efforts, most systems have not recovered – particularly in the marine realm, where between 50–90% of ecosystems remain in an altered state [62]. This highlights the need for active intervention for the recovery of degraded systems [63]. Most of the theory and examples of restoration come from terrestrial systems [64]. In contrast, restoration is much less prevalent in marine systems, where most rehabilitation is done in soft sediment habitats with seagrasses, saltmarshes and mangroves, or on coral reefs [65]–[67]. Kelp forests have received much less attention, despite being neither less impacted nor at less risk of impact [68]. Some of the few seaweed restoration attempts have failed due to negative impacts of processes such as grazing and propagule supply [35], [69]. So, restoration projects can fail due to poor understanding of the ecological processes and interactions operating in those systems [68].

Our initial efforts at the restoration of *Phyllospora* in Sydney are encouraging and suggest that restoration *via* transplantation, using the methods we have described, is possible and also relatively cost-effective. ‘Re-vegetation’ of approximately one hectare of subtidal reef as described here cost *ca* AUD 38 K including materials, transport and personnel. This estimate sits towards the lower end of coral reef restoration programs (which vary between USD 13 K and 100 M per hectare) [32] and could likely be reduced through continued methods development and optimisation. The feasibility and success of restoration of this species will, however, depend on rigorous ecological research into the processes and interactions that affect recruitment and survival of *P. comosa*. Such information will provide large scale restoration programs with ecologically sensible and informed goals to successfully restore these underwater forests.

Given the value of commercial and recreational fisheries and the importance of this habitat to those industries, not to mention the aesthetic appeal of subtidal macroalgal forests to marine recreational activities, restoration costs are likely to be offset by economic and social gains. The restoration of such valuable habitat may, in turn, restore species of commercial or recreational importance and enhance local biodiversity and/or coastal productivity [70]. Macroalgal restoration programs with clearly defined goals and whose success is measured against multiple reference locations to account for natural variability [68] could enhance local biodiversity and productivity. Similar methods could be used at larger scales and with other species to ameliorate some of the ecological impacts of disappearing kelp forests and seaweed beds globally.

## Supporting Information

Table S1 Pairwise comparisons of slopes of survival curves of algae that originated from (a) Palm Beach or (b) Cronulla among treatments (U, D, TL, TP) in the first experiment, using *F*-tests (*df*: 1, 6).
(DOCX)

Table S2 Analysis of survival (%) of transplanted *Phyllospora* from different donor populations at the recipient sites five months after the second experiment. Donor was fixed with 2 levels (TP from Cronulla or Palm Beach), Place of destination was random with 2 levels (Cape Banks and Long Bay). Replicates were the 0.25 m^2^ plots (*n* = 4). Cochran's test for homogeneity of variances: *C* = 0.32 ns. Non-significant terms with *P*>0.25 were pooled.
(DOCX)

Table S3 Analysis of photosynthetic quantum yield of Phyllospora (n = 3) three months after the first experimental transplant. Treatment was fixed with 4 levels (U, D, TL, TP), Place of origin was random with 2 levels (Cronulla, Palm Beach). Cochran's test for homogeneity of variances: C = 0.23 ns.
(DOCX)

Table S4 Analyses of % cover of bleaching on *Phyllospora* at the end of the (a) first and (b) second experimental transplants. Treatment was fixed with 4 levels (a: U, D, TL, TP; b: U, TL, TP-LB, TP-CB), Place of origin was random with 2 levels (Cronulla, Palm Beach). Replicates were the *Phyllospora* (a: *n* = 3; b: *n* = 7). Cochran's test for homogeneity of variances: a: *C* = 0.25 ns; b: *C* = 0.71, *P*<0.01.
(DOCX)

Table S5 Analysis of % cover of epibiota on *Phyllospora* (*n* = 7) five months after the second experimental transplant. Treatment was fixed with 4 levels (U, TL, TP-LB, TP-CB), Place of origin was random with 2 levels (Cronulla, Palm Beach). Cochran's test for homogeneity of variances: *C* = 0.95, *P*<0.01.
(DOCX)

Table S6 Analyses of lengths of recruits at several distances from restored (R) or reference (Ref) patches of *Phyllospora* six months after the start of the second experiment. Distance was fixed with 2 levels (inside, edge), R vs Ref was fixed with 2 levels, Place was random nested in RvRef (1 level for R, 2 for Ref), Patch was random nested in Place (1 level for R, 2 for Ref). Lengths of recruits were averaged per quadrat (*n* = 5). Data were square-root(X+1) transformed to make variances homogeneous. Cochran's test for homogeneity of variances: *C* = 0.31 ns. Non-significant terms with *P*>0.25 were pooled.
(DOCX)
