# Supplementary material for: Towards Restoration of Missing Underwater Forests
Source: PLoS One. 2014 Jan 8;9(1):e84106. doi: 10.1371/journal.pone.0084106 (PMC3885527; doi:10.1371/journal.pone.0084106)
Supplement: Table S1 — Pairwise comparisons of slopes of survival curves of algae that originated from (a) Palm Beach or (b) Cronulla among treatments (U, D, TL, TP) in the first experiment, using F-tests (df: 1, 6). (DOCX) [file pone.0084106.s001.docx]

**Table S1** Pairwise comparisons of slopes of survival curves of algae that originated from (a) Palm Beach or (b) Cronulla among treatments (U, D, TL, TP) in the first experiment, using *F*-tests (*df*: 1, 6).

|  | (a) Palm Beach | | | (b) Cronulla | | |
| --- | --- | --- | --- | --- | --- | --- |
|  | U | D | TL | U | D | TL |
| D | *F* = 21.45 ***P* = 0.04** |  |  | *F* = 2.09 *P* = 0.20 |  |  |
| TL | *F* = 4.56 *P* = 0.08 | *F* = 3.20 *P* = 0.12 |  | *F* = 11.26 ***P* = 0.02** | *F* = 0.66 *P* = 0.45 |  |
| TP | *F* = 26.77 ***P* < 0.01** | *F* = 6.61 ***P* = 0.04** | *F* = 0.04 *P* = 0.85 | *F* = 0.01 *P* = 0.92 | *F* = 1.95 *P* = 0.21 | *F* = 10.85 ***P* = 0.02** |
