# Supplementary material for: Towards Restoration of Missing Underwater Forests
Source: PLoS One. 2014 Jan 8;9(1):e84106. doi: 10.1371/journal.pone.0084106 (PMC3885527; doi:10.1371/journal.pone.0084106)
Supplement: Table S2 — Analysis of survival (%) of transplanted Phyllospora from different donor populations at the recipient sites five months after the second experiment. Donor was fixed with 2 levels (TP from Cronulla or Palm Beach), Place of destination was random with 2 levels (Cape Banks and Long Bay). Replicates were the 0.25 m2 plots (n = 4). Cochran's test for homogeneity of variances: C = 0.32 ns. Non-significant terms with P>0.25 were pooled. (DOCX) [file pone.0084106.s002.docx]

**Table S2** Analysis of survival (%) of transplanted *Phyllospora* from different donor populations at the recipient sites five months after the second experiment. Donor was fixed with 2 levels (TP from Cronulla or Palm Beach), Place of destination was random with 2 levels (Cape Banks and Long Bay). Replicates were the 0.25 m^2^ plots (*n* = 4). Cochran’s test for homogeneity of variances: *C* = 0.32 ns. Non-significant terms with *P* > 0.25 were pooled.

| Source | *df* | MS | *F* | *P* |
| --- | --- | --- | --- | --- |
| Donor | 1 | 900 | 1.86 | 0.20 |
| Place of destination | 1 | 400 | 0.83 | 0.38 |
| Do x Pl | 1 | 100 | Pooled |  |
| Residual | 12 | 517 |  |  |
