# Supplementary material for: Towards Restoration of Missing Underwater Forests
Source: PLoS One. 2014 Jan 8;9(1):e84106. doi: 10.1371/journal.pone.0084106 (PMC3885527; doi:10.1371/journal.pone.0084106)
Supplement: Table S3 — Analysis of photosynthetic quantum yield of Phyllospora (n = 3) three months after the first experimental transplant. Treatment was fixed with 4 levels (U, D, TL, TP), Place of origin was random with 2 levels (Cronulla, Palm Beach). Cochran's test for homogeneity of variances: C = 0.23 ns. (DOCX) [file pone.0084106.s003.docx]

**Table S3** Analysis of photosynthetic quantum yield of *Phyllospora* (*n* = 3) three months after the first experimental transplant. Treatment was fixed with 4 levels (U, D, TL, TP), Place of origin was random with 2 levels (Cronulla, Palm Beach). Cochran’s test for homogeneity of variances: *C* = 0.23 ns.

| Source | *df* | MS | *F* | *P* |
| --- | --- | --- | --- | --- |
| Treatment | 3 | 0.01 | 2.25 | 0.76 |
| Place | 1 | 0.00 | 0.80 | 0.38 |
| Tr x Pl | 3 | 0.03 | 5.48 | **<0.01** |
| Residual | 16 | 0.01 |  |  |
| SNK | From Cronulla: U = D = TL = TP-LB  From Palm Beach: U = D > TL = TP-CB | | | |
