# Supplementary material for: Towards Restoration of Missing Underwater Forests
Source: PLoS One. 2014 Jan 8;9(1):e84106. doi: 10.1371/journal.pone.0084106 (PMC3885527; doi:10.1371/journal.pone.0084106)
Supplement: Table S4 — Analyses of % cover of bleaching on Phyllospora at the end of the (a) first and (b) second experimental transplants. Treatment was fixed with 4 levels (a: U, D, TL, TP; b: U, TL, TP-LB, TP-CB), Place of origin was random with 2 levels (Cronulla, Palm Beach). Replicates were the Phyllospora (a: n = 3; b: n = 7). Cochran's test for homogeneity of variances: a: C = 0.25 ns; b: C = 0.71, P<0.01. (DOCX) [file pone.0084106.s004.docx]

**Table S4** Analyses of % cover of bleaching on *Phyllospora* at the end of the (a) first and (b) second experimental transplants. Treatment was fixed with 4 levels (a: U, D, TL, TP; b: U, TL, TP-LB, TP-CB), Place of origin was random with 2 levels (Cronulla, Palm Beach). Replicates were the *Phyllospora* (a: *n* = 3; b: *n* = 7). Cochran’s test for homogeneity of variances: a: *C* = 0.25 ns; b: *C* = 0.71, *P* < 0.01.

|  | (a) Autumn | | | | (b) Spring | | | | |
| --- | --- | --- | --- | --- | --- | --- | --- | --- | --- |
| Source | *df* | MS | *F* | *P* | *df* | MS | | *F* | *P* |
| Treatment | 3 | 29 | 0.10 | 0.38 | 3 | 255 | | 4.69 | 0.12 |
| Place | 1 | 84 | 0.80 | 0.96 | 1 | 126 | | 11.63 | **<0.01** |
| Tr x Pl | 3 | 307 | 2.90 | 0.07 | 3 | 54 | | 5.02 | **<0.01** |
| Residual | 16 | 105 |  |  | 48 | 11 | |  |  |
| SNK |  |  | | |  | | From Cronulla:  TP-LB > U = TL = TP-CB  From Palm Beach:  TP-LB > TL > U = TP-CB | | |
