# Supplementary material for: Towards Restoration of Missing Underwater Forests
Source: PLoS One. 2014 Jan 8;9(1):e84106. doi: 10.1371/journal.pone.0084106 (PMC3885527; doi:10.1371/journal.pone.0084106)
Supplement: Table S5 — Analysis of % cover of epibiota on Phyllospora (n = 7) five months after the second experimental transplant. Treatment was fixed with 4 levels (U, TL, TP-LB, TP-CB), Place of origin was random with 2 levels (Cronulla, Palm Beach). Cochran's test for homogeneity of variances: C = 0.95, P<0.01. (DOCX) [file pone.0084106.s005.docx]

**Table S5** Analysis of % cover of epibiota on *Phyllospora* (*n* = 7) five months after the second experimental transplant. Treatment was fixed with 4 levels (U, TL, TP-LB, TP-CB), Place of origin was random with 2 levels (Cronulla, Palm Beach). Cochran’s test for homogeneity of variances: *C* = 0.95, *P* < 0.01.

| Source | *df* | MS | *F* | *P* |
| --- | --- | --- | --- | --- |
| Treatment | 3 | 22686 | 414.80 | **<0.01** |
| Place | 1 | 16 | 0.22 | 0.64 |
| Tr x Pl | 3 | 54 | 0.74 | 0.53 |
| Residual | 48 | 74 |  |  |
| SNK | TP-CB > U = TL = TP-LB | | | |
