# Supplementary material for: Towards Restoration of Missing Underwater Forests
Source: PLoS One. 2014 Jan 8;9(1):e84106. doi: 10.1371/journal.pone.0084106 (PMC3885527; doi:10.1371/journal.pone.0084106)
Supplement: Table S6 — Analyses of lengths of recruits at several distances from restored (R) or reference (Ref) patches of Phyllospora six months after the start of the second experiment. Distance was fixed with 2 levels (inside, edge), R vs Ref was fixed with 2 levels, Place was random nested in RvRef (1 level for R, 2 for Ref), Patch was random nested in Place (1 level for R, 2 for Ref). Lengths of recruits were averaged per quadrat (n = 5). Data were square-root(X+1) transformed to make variances homogeneous. Cochran's test for homogeneity of variances: C = 0.31 ns. Non-significant terms with P>0.25 were pooled. (DOCX) [file pone.0084106.s006.docx]

**Table S6** Analyses of lengths of recruits at several distances from restored (R) or reference (Ref) patches of *Phyllospora* six months after the start of the second experiment. Distance was fixed with 2 levels (inside, edge), R vs Ref was fixed with 2 levels, Place was random nested in RvRef (1 level for R, 2 for Ref), Patch was random nested in Place (1 level for R, 2 for Ref). Lengths of recruits were averaged per quadrat (*n* = 5). Data were square-root(X+1) transformed to make variances homogeneous. Cochran’s test for homogeneity of variances: *C* = 0.31 ns. Non-significant terms with *P* > 0.25 were pooled.

| Source | *df* | MS | *F* | *P* |
| --- | --- | --- | --- | --- |
| Distance | 1 | 1.56 | 2.15 | 0.15 |
| R vs Ref | 1 | 0.31 | 0.04 | 0.87 |
| Place(RvRef) | 1 | 11.69 | 9.70 | 0.09 |
| D x RvRef | 1 | 1.89 | 2.61 | 0.11 |
| Patch(Pl(RvRef)) | 2 | 1.21 | 1.67 | 0.20 |
| D x Pl(RvRef) | 1 | 0.07 | Pooled |  |
| D x Pa(Pl(RvRef)) | 2 | 0.61 | Pooled |  |
| Residual | 40 | 0.75 |  |  |
